# Patterns of Pre-pregnancy Care Usage among Reproductive Age Women in Kedah, Malaysia

**Published:** 2018-11

**Authors:** Rozaimah ABU TALIB, Idayu Badilla IDRIS, Rosnah SUTAN, Norizan AHMAD, Norehan ABU BAKAR

**Affiliations:** 1. Kerian Health District, Parit Buntar, Perak, Malaysia; 2. Dept. of Community Health, Faculty of Medicine, Universiti Kebangsaan Malaysia, Kuala Lumpur, Malaysia; 3. Family Health Development Division, Health Department of Kedah, Alor Star, Kedah, Malaysia; 4. Bandar Baharu District Health Office, Kedah, Malaysia

**Keywords:** Pre-pregnancy care services, Usage, Determinant

## Abstract

**Background:**

This cross-sectional was aimed to assess the prevalence of pre-pregnancy care services usage and its determinant factors among women of reproductive age in Kedah, Malaysia.

**Methods:**

Overall, 1347 respondents who attended 24 government health clinics, were chosen using systematic multistage random sampling. A validated self-administered questionnaire which consisted of sections including socio-demographic characteristics, social support, knowledge on pre-pregnancy care, perception on risk of pregnancy, health status, as well as intention and awareness on pre-pregnancy care services were distributed.

**Results:**

The prevalence of utilization of pre-pregnancy care services was still low i.e. 44.0%. Bivariate and multivariate analysis showed consistent significant level between all factors and pre-pregnancy care usage except for family planning practice. The factors that showed significant difference with the usage of pre-pregnancy care services were age of more than 35 (*P*<0.001), high education level (*P*<0.001), non-working mothers (*P*<0.001), multipara (*P*=0.001), awareness on the existence of pre-pregnancy care services in government health facilities (*P*<0.001), intention to use the services (*P*=0.0030), having medical illness (*P*=0.005), having social support (*P*=0.001), high knowledge (*P*<0.001), and positive perception (*P*<0.001).

**Conclusion:**

Low usage of pre-pregnancy care services can be improved through health screening on reproductive-aged women with positive determinant factors at the triage level in integrated clinics. Information and knowledge on pre-pregnancy services should be disseminated among community members through various means including roadshows and pre-wedding workshops.

## Introduction

Today, the continued increase in the burden of maternal and infant mortality has become a public health concern globally. Worldwide, over 350000 women of reproductive age die each year from complications of pregnancy and during delivery, while more than 15 million women suffer from long-term illnesses or disability (1). The risk of unfavorable pregnancy outcomes is higher in developing and less developed countries compared to developed countries (2).

In addition to the burden of maternal mortality and morbidity rate which showed increasing trend in developing and less developed countries, globally, in which each year about 3.6 million newborns die within the first four weeks after birth (3). Similarly, maternal health complications contributed to at least 1.5 million neonatal deaths in the first week of delivery and 1.4 million fetuses die in utero or termed as “stillborn babies” (1). Worldwide, among the main causes of neonatal deaths were premature births, low birth weight babies and babies with congenital abnormalities (4). Under-weight mothers and maternal complications also lead to a high risk of infant mortality (3).

In Malaysia, although the mortality rate among women of reproductive ages has reduced over the years, this reduction has been almost stagnant for the past ten years (27.9 per 100000 live birth in 2005 to 25.2 per 100000 live birth in 2013) (5). The fact that the fifth Millennium Development Goal (MDG 5) which had not fully achieved its target especially in developing and less developed countries, should be given serious attention. At present, the new Sustainable Development Goal (SDG) has been introduced mainly with the aim to reduce poverty as well as to overcome inequality and injustice. In order to achieve the broader aim of SDG, several measures need to be undertaken. One of the strategies includes a proper implementation of pre-pregnancy care (PPC) services for the reproductive age group of women. Besides, intervention before pregnancy can improve the pregnancy outcome for both babies and mothers (6).

Center of Diseases Control and Prevention (CDC) 2006 defines pre-pregnancy care as “a set of intervention that aims to identify and modify biomedical risk, behavioral and social risks to a women’s health through prevention and treatment” (7–8). Pre-pregnancy care services in the state of Kedah, Malaysia is one of the strategies to reduce maternal deaths caused by chronic diseases as stated in the plan of action to achieve the fifth Millennium Development Goal in this state (9). As early as in the 80s, pre-pregnancy care can reduce the incidence of adverse pregnancy outcomes such as major defects in diabetic patients (10). Today, there are sufficient scientific evidence to prove that these interventions which are carried out before pregnancy have the ability to reduce the adverse effects of pregnancy (11).

Although pre-pregnancy care services had been proven to be successful, the level of utilization of these services and the knowledge of Malaysian women in the reproductive ages about pre-pregnancy care and its services remains unclear. Many women in this country including those from the state of Kedah do not use pre-pregnancy care services, which are readily available and affordable. If these diseases were not controlled promptly, it will result in adverse outcome to the mother as well as the baby (12–13). The prevalence of pre-pregnancy care services in Philadelphia is relatively high (52%). Even though the usage of pre-pregnancy care services was quite high in this part of the world, there were still many barriers to women’s reproductive age in using such services such as from the social and cultural point of view (14).

We aimed to determine the prevalence of pre-pregnancy care services usage and its determinant factors among women of reproductive ages in Kedah, Malaysia.

## Methods

This cross-sectional study was carried out among married reproductive-aged women between the ages of 18 to 45 year old from all ethnic groups in Kedah in May 2015. It was conducted in 12 districts in Kedah chosen through a multistage random sampling. Eight districts were chosen and three government health clinics were then selected from each district. Overall, 24 government health clinics were finally selected to be included in this study. Selection of the respondents were through systematic random sampling where it was based on registration number listed in the registration book at the integrated counter in the 24 government health clinics involved in this study. Every five women who fulfilled the inclusion criteria were chosen to avoid bias.

We used a validated self-administered questionnaire which had satisfactory value of Cronbach’s alpha of 0.927. This questionnaire consisted of 7 domain sections including socio-demographic characteristics, socio-economic background, social support, and knowledge on pre-pregnancy care, perception on risk of pregnancy, health status, as well as intention and awareness on pre-pregnancy care services. Overall, 1347 mothers answered the self-administered questionnaire in the Malay language in the clinics.

Six sub-questions, which consisted of 44 fractional responses, were used to assess the knowledge of pre-conception care and its services which includes knowledge of what was pre-pregnancy care, to whom such services should be designated for, the importance and goals of pre-pregnancy care services, types of services provided in the pre-pregnancy care clinics and where to get the service. Twelve items questionnaire related to pre-pregnancy care and its services were used to test the perception of respondents including perception towards risk of pregnancy as well as perception towards outcome of pregnancy and its complication. Meanwhile, in the social support section, there were four questions given to respondents.

Descriptive characteristics were summarized and presented as numbers and percentages. Chi-square test was used to examine the relationship between each variable and usage of pre-pregnancy care services. This was followed by multiple logistic regression analysis utilized to examine the associations between the significant variables (*P-*value<0.05) with the uptake of pre-pregnancy care services. Dependent variable was the usage of pre-pregnancy care services while the independent variables were the social-demographic factors, awareness, intention, knowledge, perception, social support and medical history. Data were analyzed using SPSS ver. 20.0 (Chicago, IL, USA).

Ethical approvals were obtained from National Medical Research Registration and Kedah State Health Department as well as Biomedical Ethics Committee UKM Medical Center.

## Results

A total of 1500 questionnaires and consent forms were given to all respondents. However, only 1347 respondents agreed to participate in this study giving a response rate of 90%. Table 1 shows the socio-demographic characteristics of the respondents.

**Table 1:** Socio-demographic profile (n=1347)

***Variable***		***Number***	***%***
Age group (yr)	<35	791	58.7
	≥35	556	41.3
Mean age (SD)	32 (+6.377)		
Ethnicity	Malay	1190	88.3
	Non-Malay	157	11.7
Education level	High (college and higher)	345	25.6
	Low (secondary to lower)	1002	74.4
Occupational	Not working	601	44.6
	Working	746	55.4
House whole income	< 259 USD	483	35.9
	≥ 259 USD	864	64.1
Mean (SD)	560 USD (+ 363)		
Parity	Multipara	1083	80.4
	Nulliparous	264	19.6
Family planning User	Yes	540	40.1
	No	807	59.9
Pregnant	Yes	407	30.2
	No	940	69.8
Planned pregnancy (n=940)	Yes	540	40.1
	No	807	59.9
Distance from health facilities	<5 KM	573	42.5
	≥5 KM	774	57.5

Most of the respondents were aware and they had intention to use the services. However, only 44.0% had ever used pre-pregnancy care services. Overall, the level of knowledge about pre-conception care and its services was low and respondents had negative perception on pre-conception care and its services. Most of the respondents had good social support in utilizing pre-pregnancy care services (Table 2).

**Table 2:** Awareness, intention and the use of pre-pregnancy care services, knowledge, perception, social support and medical history (n=1347)

***Variable***		***Number (n)***	***Percentage (%)***
Awareness on PPC services	Yes	1104	82.0
	No	243	18.0
Intention to used PPC	Yes	1147	85.2
	No	200	14.8
PPC Usage	Yes	593	44.0
	No	754	56.0
Knowledge	High (score ≥22)	647	48.0
	Low (Score <22)	700	52.0
Perception	Positive (Score ≥31)	637	47.4
	Negative (Score <31)	709	52.6
Social support	Yes (Score ≥2)	731	54.3
	No (Score <2)	616	45.7
Medical history	Yes	698	51.8
	No	649	48.2

### Bivariate analysis

Table 3 shows that only older age, high education level, non-working mothers, multiparous mothers and previous usage of family planning practice had a significant relationship with the use of pre-pregnancy care services. In Table 4, using bivariate analysis, women who had an awareness of the existence of pre-pregnancy care services, those who had a high level of knowledge (score more than 22), women with positive perception, good social support and women with a history of chronic diseases were more likely to utilize pre-pregnancy care services.

**Table 3:** Relationship between socio-demographic and socio-economic with the use of pre-pregnancy care services, n=1347

***Characteristic***	***PPC*** ***usage***		***Not PPC usage***		***X^2^***	***P value***
Age (yr)	***n***	***%***	***n***	***%***		
≥35	290	52.2	266	47.8	25.424	<0.001^*^
<35	303	38.3	488	61.7		
Ethnicity							
Malay	520	43.7	670	56.3	0.441	0.507
Non-Malay	73	46.5	84	53.5		
Education level							
High ^a^	177	51.3	168	48.7	9.976	0.002^*^
Low ^b^	416	41.5	586	58.7		
Occupation							
Not working	310	51.6	291	48.4	25.148	<0.001^*^
Working	283	37.9	463	62.1		
House whole income							
≥ RM 1660	369	42.7	495	57.3	1.692	0.193
< RM 1660	224	46.4	259	53.6		
Parity							
Multipara	545	50.3	538	49.7	88.982	<0.001^*^
Nulliparous	48	18.2	216	81.8		
Family planning user							
Yes	262	48.5	278	51.5	7.389	0.007^*^
No	331	41.0	476	59.0		
Pregnant							
Yes	173	42.5	234	57.5	0.545	0.460
No	420	44.7	520	55.3		
Planned pregnancy (n=940)							
Yes	163	45.9	192	54.1	0.352	0.553
No	257	43.9	328	56.1		
Distance from health facilities							
<5 KM	267	46.6	306	53.4	2.679	0.102
≥5 KM	326	42.1	448	57.9		

*X*^*2*^ – Chi-square,

* significant *P*-value<0.05,

a College or University,

b secondary school and below

**Table 4:** The relationship between awareness, intention, knowledge level, perception, social support and underlying medical illness with the use of pre-pregnancy care services

***Characteristics***	***PPC usage***		***Not PPC usage***		***X^2^***	***P-value***
**Awareness**							
Yes	571	51.7	533	48.3	147.134	<0.001^*^
No	22	9.1	221	90.1		
**Intention**							
Yes	566	49.3	581	50.7	88.801	0.001^*^
No	27	13.5	173	86.5		
**Knowledge**							
High	400	61.8	247	38.2	160.076	<0.001^*^
Low	193	27.6	507	72.4		
**Perception**							
Positive	408	63.9	230	36.1	195.297	<0.001^*^
Negative	185	26.1	542	73.9		
**Social support**						
Yes	380	52.0	351	48.0	41.098	0.001^*^
No	213	34.6	403	65.4		
**Medical history**						
Yes	399	52.0	368	48.0	46.228	0.001^*^
No	194	33.4	386	66.6		

X^*2*^ – Chi square,

* significant *P-*value <0.05

### Multivariate analysis

Multiple logistic regression analysis was finally utilized to determine the factors significantly associated with pre-pregnancy care users. Reproductive aged women of more than 35 years, women with higher level of education, non-working women and multiparous women had higher odds of using the pre-pregnancy care services compared to others. In addition, women who were aware of the existence of pre-pregnancy care services at government clinics and hospitals and women who had an intention to use pre-pregnancy care services had higher odds to use the services. Women with chronic medical illness or had bad obstetrics history, women with good social support, women who had high knowledge and positive perception, had higher odds of using pre-pregnancy care services as shown in Table 5.

**Table 5:** Determinant of pre-pregnancy care usage by univariable and multivariable logistic regression

***Characteristic***	***PPC usage n(%)***	***Non PPC usage n(%)***	***COR^a^ (CI 95%)***	***AOR^b^ (CI 95%)***	***Wald***	***P-value***
**Age (yr)**						
≥35	290(52.2)	266(47.8)	1.76	3.06	33.67	<0.001
[<35]	303(38.3)	488(61.7)	(1.41–2.19)	(2.10–4.46)		
**Education level**						
High ^a^	177(51.3)	168(48.7)	1.16	3.68		
[Low^b^]	416(41.5)	586(58.7)	(1.16–1.90)	(2.40–5.65)	35.79	<0.001
**Occupational**						
Not working	310(51.6)	291(48.4)	1.74	1.95	12.60	0.001
[Working]	283(37.9)	463(62.1)	(1.40–2.17)	(1.35–2.82)		
Pariti						
Multipara	545(50.3)	538(49.7)	4.56	2.78		
[Nulliparous]	48(18.2)	216(81.8)	(3.26–6.37)	(1.68–4.60)	15.85	0.001
**Awareness**						
Yes	571(51.7)	533(48.3)	10.76	5.18	29.90	<0.001
[No]	22(9.1)	221(90.9)	(6.84–16.94)	(2.87–9.34)		
Intention												
Yes	566(49.3)	581(50.7)	6.24	1.94	4.68	0.030
[No]	27(13.5)	173(86.5)	(4.09–9.52)	(1.07–3.55)		
**Medical History**						
Yes	399(52.0)	368(48.0)	2.16	1.64		
[No]	194(33.4)	386(66.6)	(1.73–2.70)	(1.16–2.33)	7.84	0.005
**Social support**						
Yes	399(57.0)	301(43.0)	2.05	1.30		
[No	194(30.0)	453(47.0)	(1.64–2.55)	(1.13–1.50)	13.60	0.001
Knowledge						
High	308(77.6)	89(22.4)	5.94	1.18	50.15	<0.001
[Low]	92(36.8)	158(63.2)	(4.19–8.42)	(1.15–1.21)		
Perception												
Positive	100(41.5)	141(58.5)	2.80	1.17	22.02	<0.001
[Negative]	93(20.3)	366(79.5)	(1.98–3.93)	(1.14–1.21)		

Degree of freedom (df) =1, [ ]-reference group,

a College and above,

b secondary school and below

## Discussion

Our study found that the prevalence of pre-pregnancy care usage among reproductive-aged women in Kedah was considerably low, i.e. 44% only. Our findings were consistent with findings from a meta-analysis research, which found that the prevalence of pre-pregnancy care services among women who had diabetes was low, i.e. between 34%–38% only (15). Another research on the use of pre-pregnancy care services among women with chronic diseases also found that only 18.1% to 45% women used these services. (16).

In terms of education, we found, women who had a higher level of education were four times more likely to use pre-pregnancy care services. These study’s findings were also consistent with few other studies where they also found women with higher education were more likely to use such services (17–18). Women who had higher education were more aware of the importance of pre-pregnancy health care than women with lower education.

We also found the use of family planning had a significant association with pre-pregnancy care services utilization. However, after conducting multivariate analysis it was found that there was no significant association between practicing family planning with the use of such services. In this study, only 40.1% of women were using family planning which was almost similar to findings in another study in Kedah i.e. 43.0% (19). However, both percentages were lower than the result obtained from National Population and Family Development Board (NPFDB), Malaysia in 2004 i.e. 51.7% (20, 28). Another study carried out in the state of Selangor found only 19.2% high-risk women practiced contraception (21). Our study also showed majority of the respondents were aware of the existence of pre-pregnancy care services and this result was significant. These findings were similar to study conducted in United Kingdom among women with diabetes in which they found 90% of women were aware of the existence of pre-pregnancy care services (22).

However, in southern Nigeria, only 65.9.1% had ever heard of pre-pregnancy care (23).

In terms of intention to use pre-pregnancy care services, we found the majority of respondents had intention to use the pre-pregnancy care services. Our study findings were consistent with the study carried out in the United States (24). Another study conducted among women of reproductive age-group found most of them had intention to undergo pre-pregnancy screening for cystic fibrosis disease. However, according to this study, only 56% finally wanted to carry out pre-pregnancy screening for cystic fibrosis (25).

Our findings also showed that there was a significant relationship between social support and the use of pre-pregnancy care services. Women who had good social support were more likely to use pre-pregnancy care services than women who had less social support. This was because support from people surrounding these women, especially husbands, ensures women to acknowledge pre-pregnancy care services and encourages them to use these services. Social support from a partner was very important in giving women the opportunity to use pre-pregnancy care services (26–28).

We also found the overall level of knowledge of reproductive women in this country with regard to pre-pregnancy care services were low. We found knowledge of pre-pregnancy care were significantly associated with the use of pre-pregnancy care services. Another study supported the findings of this study (29). A study conducted in Sri Lanka found that the population in this country had low level of knowledge on pre-pregnancy care particularly about vaccination (30). Although the women of reproductive age had chronic diseases, the majority of them had low level of knowledge about pre-pregnancy care (31).

For the perception of pre-pregnancy care and its services, the majority of women had a negative perception of pre-conception care and its services. Bivariate analysis showed a significant relationship between the perception of women’s pre-pregnancy care and use of such services. This study also found, most respondents did not agree that they were likely to become pregnant even when they were not using family planning although they were in the reproductive age. A study also supported this finding, where a lot of women in the reproductive age did not feel that they were at risk for pregnancy or had a negative perception of the risk of pregnancy even if they were not using these contraceptives (32).

In terms of health status, our analysis found that most women had at least one health problem and showed a significant association between health problems with the use of pre-pregnancy care services. Health history involves chronic health problems or gynecological problems as well as problems during pregnancy. Almost 70% of women who had pre-pregnancy screening in health clinics in Selangor had at least one underlying medical history (21). Those who had chronic diseases were more likely to use pre-pregnancy care services more than those who did not have any type of chronic diseases (27).

We also found multiparous women had three times possibility of using pre-pregnancy care services compared to those who never had any children. However, multiparous women used less pre-pregnancy care services compared to nulliparous women. This was because they felt that they had enough knowledge on pregnancy as well as having had negative experiences during previous pregnancies (15, 33).

The low prevalence of pre-pregnancy care services uptake in Kedah, Malaysia was most likely due to lack of promotion and awareness about the importance of pre-pregnancy care which has been proven to be important to all reproductive women especially those who had underlying medical illnesses. We should strive to increase the knowledge and usage of these services among such women mainly because many studies had shown a significant decrease in adverse pregnancy outcome including maternal mortality i.e. from 15%–35%, reduction of perinatal mortality by 66% and decrease congenital malformation from 7.4% to 1.9% (35) due to these services.

## Conclusion

It is very important to improve the knowledge and perception of pre-conception care and services among women of reproductive age in this country, considering the low utilization of pre-pregnancy care services as shown in this study. With the effort by multiple stakeholders taken to improve the knowledge and perception of reproductive-aged women on such services, especially among women who are younger, less educated and do not have children, may result in the reduction of poor pregnancy outcome. This include the reduction of maternal mortality rate and at the same time, we hope to achieve one of the SDG targets in 2030, i.e. to reduce maternal mortality rate to 8.7 per 100,000 live births.

## Ethical considerations

Ethical issues (Including plagiarism, informed consent, misconduct, data fabrication and/or falsification, double publication and/or submission, redundancy, etc.) have been completely observed by the authors.
